# Dystonic Tremor as an Atypical Presentation of Cervical Radiculopathy

**DOI:** 10.7759/cureus.51441

**Published:** 2024-01-01

**Authors:** Eric Chun-Pu Chu, Lucina Ng

**Affiliations:** 1 Chiropractic and Physiotherapy Centre, New York Medical Group, Hong Kong, CHN

**Keywords:** chiropractor, tremor, dystonia, cervical radiculopathy, chiropractic, brachialgia

## Abstract

Cervical radiculopathy often presents with neck pain, sensory disturbances, or motor weakness. This case report describes an atypical presentation of cervical radiculopathy manifesting as dystonic tremor and brachialgia, which has been documented only once previously. A 46-year-old, right-handed, male delivery worker presented with severe neck pain that gradually evolved into a dystonic tremor and sharp aching pain across his right shoulder. Despite medical intervention, his symptoms persisted, leading him to seek chiropractic care. Physical examination and diagnostic tests revealed degenerative osteophytes, causing bilateral foraminal impingement and narrowing. As orthopedic interventions only provide temporary relief, the patient opted for conservative chiropractic management, which led to a remarkable reduction in pain and complete resolution of the dystonic tremor and brachialgia. This case demonstrates that cervical disc prolapse may manifest with dystonic tremor due to excruciating radiculopathy. Additionally, it emphasizes the potential benefits of chiropractic care in managing such atypical presentations and underscores the need for further research on the mechanisms and management of such cases.

## Introduction

Cervical radiculopathy is a neurological condition characterized by nerve root dysfunction due to compression or irritation in the cervical spine [1]. This condition typically presents as radicular pain, sensory disturbances, or motor weakness in the distribution of the affected nerve root [1]. However, atypical presentations can occur and complicate diagnostic and treatment processes [2,3].

Tremor and dystonia are two common movement disorders that may coexist or manifest separately [4]. Tremor is a rare occurrence following a neck injury, and its underlying physiological mechanism remains unclear [5]. Cervical joint instability is caused by cervical dystonia, an isolated dystonia of the cervical muscles [6]. Dystonic tremor is an unusual manifestation of cervical radiculopathy [7]. It is characterized by involuntary muscle contractions that cause abnormal postures or movements in parts of the body [7].

This case report describes the case of a 46-year-old man who presented with severe neck pain that gradually manifested as a dystonic tremor and sharp aching pain throughout his right shoulder, a constellation of symptoms associated with cervical radiculopathy. This case highlights the potential for atypical presentations of cervical radiculopathy, the diagnostic and treatment challenges they pose, and the potential benefits of chiropractic care in their management.

## Case presentation

A 46-year-old, right-hand-dominant delivery driver presented to our chiropractic clinic with a 10-day history of severe, progressive neck pain. The patient reported experiencing sudden-onset neck pain while at work, which was initially rated 8/10 in severity on a numerical pain scale. This pain disrupted his ability to drive and perform his occupational duties, such as loading packages. Over the first week, the pain worsened and became constant. Approximately five days after the initial pain onset, the patient noticed the development of dystonic posturing in the affected area. Therefore, the dystonic posturing manifested around five days after the initial pain onset. Additionally, the pain spread to the right shoulder and arm, leading to radicular symptoms affecting the right upper extremity.

**Figure 1:**
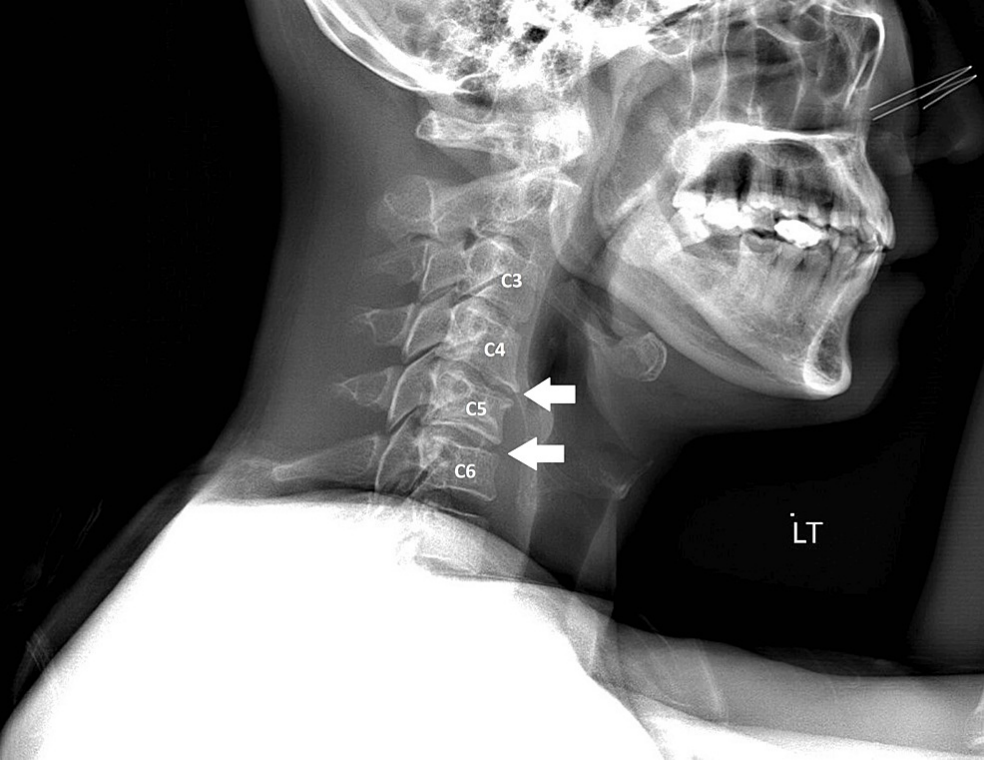
Cervical radiograph. Cervical radiograph demonstrating multilevel degenerative changes. There are anterior marginal osteophytes noted along the cervical spine. Intervertebral disc space narrowing is present at levels C2–C6, indicative of disc degeneration. Of note, the greatest degree of disc space narrowing is seen at C4/5 and C5/6, corresponding to the level of nerve root impingement seen on magnetic resonance imaging of the patient’s cervical. These degenerative changes involving the C5/6 segment align with the patient’s clinical presentation of right C6 radiculopathy.

Before his visit, he consulted his general practitioner, and cervical radiography identified cervical degenerative joint disease at the C5/6 level (Figure 1). The individual had controlled hypertension but an otherwise unremarkable past medical history. He denied any recent trauma or injury. Prior medical treatment included a trial of 300 mg gabapentin thrice daily and ibuprofen 400 mg as needed, without relief of symptoms. Due to intractable pain, rated 10/10 in severity on presentation, the patient had been unable to work for 10 days before the chiropractic consultation.

The patient presented to the chiropractic clinic with a dystonic posture, holding his neck with his right hand, and exhibiting a 5-degree right lateral flexion of the neck. Dystonic tremors with inconsistent patterns were identified in the right rotator cuff muscles. Specifically, these tremors are a type of involuntary, rhythmic muscle contractions that result in twisting, jerky movements of the right shoulder. The frequency and intensity of tremors were exacerbated during elbow extension and wrist extension. The tremors could have affected the patient’s ability to perform routine tasks that involve the use of the shoulder joint, such as lifting objects or moving the arm in particular directions. The active range of motion of the cervical spine was significantly restricted in multiple planes. Flexion and rotational movements caused significant discomfort, with flexion limited to approximately 50% of the normal range and extension limited to approximately 60%. Right lateral cervical flexion induced severe pain, with a range of motion limited to approximately 30%. Additionally, there was a restriction of approximately 40% in the rotational range of motion. These limitations in range of motion were associated with varying degrees of pain and discomfort. During the examination, positive findings on Spurling’s test, cervical distraction test, and upper limb tension test indicated cervical nerve root compression. Motor examination revealed diminished strength in the right C5 distribution (2/5) and at the C6 and C7 levels (4/5) due to pain. Sensation and reflexes were intact. Notably, the patient presented with cold extremities and elevated blood pressure measuring 145/90 mmHg.

The restricted range of motion in the cervical spine, particularly in flexion, rotation, and lateral flexion, was closely related to the patient’s radicular symptoms and dystonic posturing. These limitations in range of motion and associated discomfort indicated mechanical compression and irritation of the cervical nerve roots, leading to radiating pain, weakness, and diminished strength in the right upper extremity. Positive findings on Spurling’s test, cervical distraction test, and upper limb tension test further supported the presence of cervical nerve root compression. The dystonic posturing observed was likely a protective response of the surrounding muscles, aimed at stabilizing the spine and relieving further compression. Additionally, the patient’s cold extremities and elevated blood pressure suggested autonomic dysregulation associated with sympathetic cervical chain irritation and compressed nerve roots.

**Figure 2:**
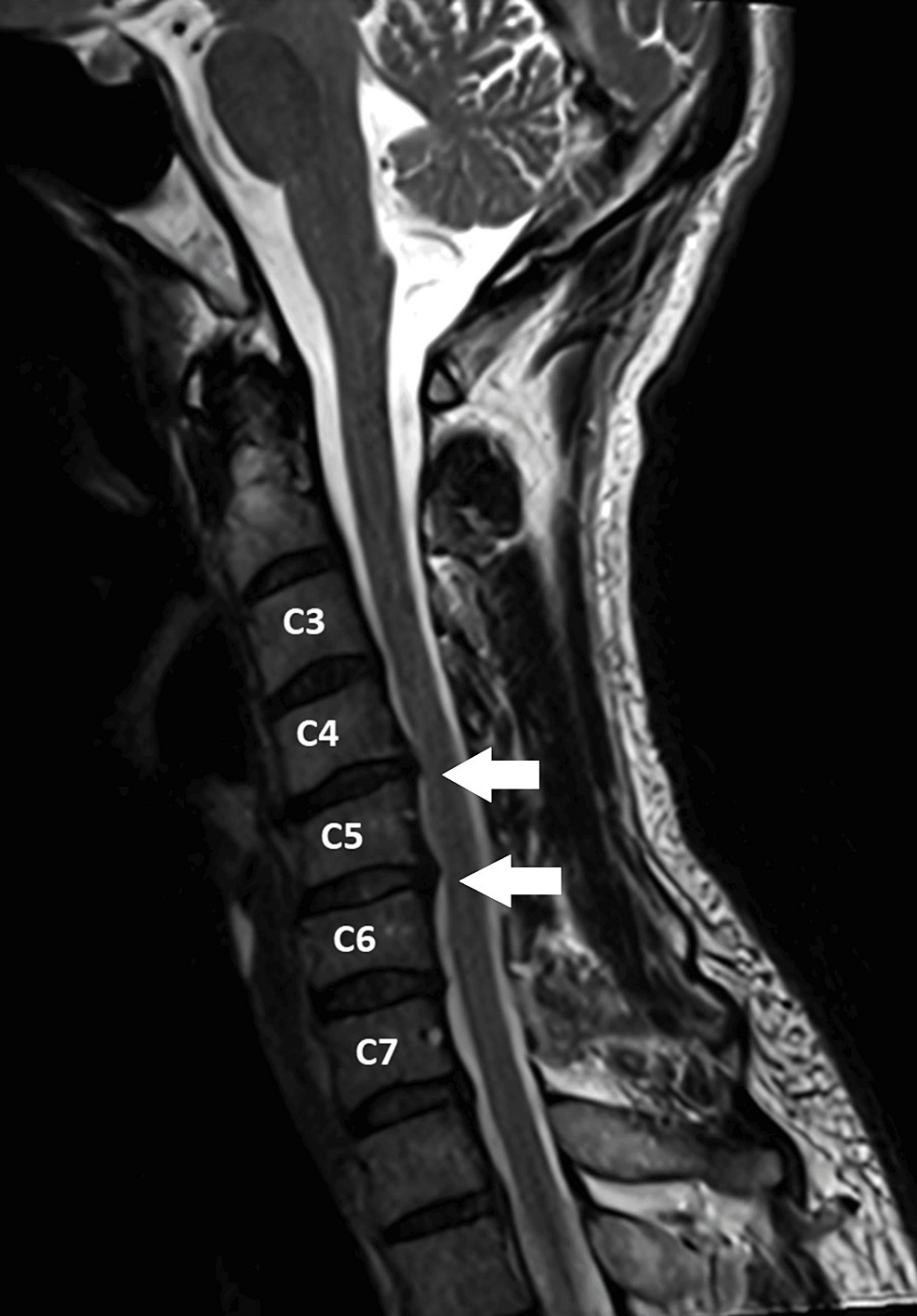
Cervical magnetic resonance image (MRI). Cervical MRI demonstrating multilevel degenerative changes and nerve root impingement. There is posterior disc bulging/prolapse from C2/3 to C7/T1, resulting in narrowing of the bilateral intervertebral foramina from C4/5 to C7/T1. The spinal cord is abutted at C4/5 and C5/C6. The right C6 nerve root impingement corresponds to the patient’s radicular symptoms in the C6 distribution.

Based on these clinical findings, the chiropractor ordered an immediate cervical magnetic resonance imaging to identify the integrity of the nervous system. The medical radiologist revealed a degenerative osteophyte complex at the C5/6 level causing impingement of the right C6 exiting nerve root (Figure 2). Furthermore, there was slight impingement observed on the bilateral C7 exiting nerve roots at the C6/7 level. Electromyography confirmed a neurogenic abnormality consistent with C6/7 radiculopathy. Notably, manual cervical distraction maneuver induced intolerable pain, prompting an immediate referral to the district hospital for specialized pain management. These diagnostic findings provided objective evidence for the compression and impingement of the cervical nerve roots, explaining the patient’s radicular symptoms and the need for further intervention.

The computed tomography-guided right C6 and C7 root block procedure was performed by the spine surgeon. The procedure involved the administration of a local anesthetic to provide targeted pain relief. The anesthetic was injected at the specific location of the right C6 and C7 nerve roots, guided by computed tomography for precise placement. This technique ensures accuracy and effectiveness in delivering the medication directly to the affected nerves. The root block procedure resulted in a comprehensive alleviation of all symptoms, including the resolution of the dystonic tremor. As a result of the successful procedure, the patient was hospitalized for seven days to closely monitor their condition and ensure pain stabilization. During this time, the pain level decreased to 7/10, and the patient’s blood pressure returned to normal. The root block procedure demonstrated its effectiveness in providing temporary relief and improving the patient’s overall well-being.

After discharge, the patient returned to the chiropractic clinic for conservative management. While chiropractic manipulative therapy can be effective in managing musculoskeletal conditions, there are potential risks and side effects such as temporary soreness or discomfort after treatment, the risk of exacerbating existing conditions, and the rare possibility of injury to the spine or nervous system. As the patient tolerated the manual cervical distraction tests, the chiropractic treatment plan included daily cervical manipulative therapy, diversified technique, for the first week alongside his existing medications. This approach led to a remarkable reduction in the pain intensity to 5/10, allowing the patient to resume his regular work schedule after the first week. Following this, the treatment frequency was decreased to thrice weekly for the subsequent three weeks, and mechanical spinal decompression therapy (Spine MTK-1, Shinhwa Medical, Busan, Republic of Korea) was incorporated into the regimen. The patient discontinued all medications during the second week of chiropractic care. This comprehensive, conservative approach ultimately led to the complete and sustained resolution of his right-sided brachialgia and dystonic tremor.

## Discussion

This case report presents an atypical manifestation of cervical radiculopathy, specifically dystonic tremor and hypertension, secondary to cervical disc prolapse and foraminal stenosis. The dystonic tremor observed in our patient is likely a consequence of the painful radiculopathy [7]. Irritation of the cervical nerve roots can disrupt inhibitory signaling and cause involuntary muscle contractions or dystonic posturing of the shoulder and neck musculature supplied by the affected nerves [8]. In addition, compression of the sensory nerve roots exacerbates muscle spasms as a protective response, further contributing to dystonia. Elevated blood pressure may also be related to autonomic dysregulation and sympathetic overactivity resulting from irritation of the sympathetic cervical chain and sympathetic fibers contained in compressed nerve roots [9]. Pain signaling itself can further increase sympathetic tone and blood pressure [10]. In summary, the dystonic tremor and hypertension observed in our patient appear to be sequelae of pathological nerve root irritation, compression, and subsequent motor and autonomic dysfunction associated with cervical radiculopathy.

The patient’s dystonia and brachialgia remained unresponsive to traditional medical interventions. Chiropractic treatment has been shown to correct cervical lordosis and improve nerve root function [11]. The restoration of autonomic nervous function can be achieved following successful cervical rehabilitation [12]. The response to chiropractic treatment, however, exemplifies the potential benefits of conservative management of cervical dystonia [13-15]. Daily cervical manipulative therapy, followed by a combination of reduced-frequency therapy and mechanical spinal decompression, resulted in the complete and sustained resolution of his symptoms. This suggests that conservative chiropractic interventions can play a vital role in managing complex cervical radiculopathy, even when initial medical and surgical interventions have failed.

Comparing this case with a similar previous case [7], it is worth noting that neither patient responded completely to medications and nerve blocks. Unlike the previously described patient, who recovered through C5/6 and C6/7 anterior cervical discectomy and fusion [7], our case was managed through chiropractic care. This suggests a potential pattern that deserves further research on the conservative and surgical management of cervical dystonic tremors.

## Conclusions

This case study emphasizes the potential for atypical presentations of cervical radiculopathy, including dystonic tremor secondary to painful brachialgia, and highlights the importance of maintaining a broad differential diagnosis. While initial medical interventions did not yield significant improvement, the patient’s remarkable response to conservative chiropractic care is noteworthy. The utilization of cervical manipulative therapy and axial mechanical spinal decompression played a crucial role in achieving a favorable outcome. This case underscores the significance of considering chiropractic interventions in the management of complex or atypical presentations of cervical radiculopathy. Furthermore, it highlights the need for further research into the mechanisms and optimal management approaches for such cases, particularly in the context of long-term outcomes.
